# 1-(2,3-Di-*O*-acetyl-4-chloro-4-de­oxy-6-*O*-tosyl-β-d-galactopyranos­yl)propan-2-one methanol 0.25-solvate

**DOI:** 10.1107/S1600536808022253

**Published:** 2008-07-19

**Authors:** Lin Yan, Feng-Wu Liu, Hong-Min Liu

**Affiliations:** a Institute of Pharmacy, Henan University, Kaifeng, Henan 475001, People’s Republic of China; b New Drug Research and Development Center, Zhengzhou University, Zhengzhou, Henan 450052, People’s Republic of China

## Abstract

The asymmetric unit of the title solvate, C_20_H_25_ClO_9_S·0.25CH_3_OH, contains one galactopyranosyl derivative and one-quarter of a methanol solvent mol­ecule. The galactopyran­ose ring is in the usual ^4^ *C*_1_ conformation, and the anomeric center of the sugar has a β configuration. The value of θ (3.44°) and the range of torsion angles [or 53.1 (5)–63.0 (5)°] reflect a slight distortion of the ^4^ *C*_1_ pyran­ose ring. A minor orientational disorder affects a carbonyl group, which was modeled with two sites for the O atom having occupancies of 0.79 (5) and 0.21 (5). The crystal studied exhibited inversion twinning.

## Related literature

For related literature, see: Lewis *et al.* (1982 ▶); Nicolaou *et al.* (1995 ▶); Paterson & Mansuri (1985 ▶); Postema (1992 ▶); Tvaroška *et al.* (2002 ▶).
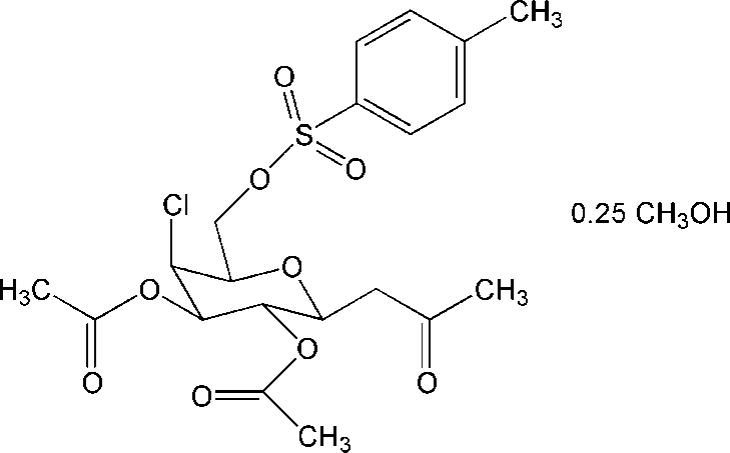


## Experimental

### 

#### Crystal data

C_20_H_25_ClO_9_S·0.25CH_4_O
*M* *_r_* = 484.92
Orthorhombic, 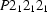

*a* = 7.3300 (15) Å
*b* = 14.608 (3) Å
*c* = 22.329 (5) Å
*V* = 2390.9 (8) Å^3^
*Z* = 4
Mo *K*α radiation
μ = 0.29 mm^−1^
*T* = 291 (2) K
0.20 × 0.17 × 0.16 mm

#### Data collection

Bruker SMART APEX CCD area-detector diffractometer
Absorption correction: multi-scan (*SADABS*; Bruker, 2001 ▶) *T*_min_ = 0.944, *T*_max_ = 0.954
7242 measured reflections
4129 independent reflections
3274 reflections with *I* > 2σ(*I*)
*R*_int_ = 0.052

#### Refinement

*R*[*F* ^2^ > 2σ(*F* ^2^)] = 0.061
*wR*(*F* ^2^) = 0.154
*S* = 1.00
4129 reflections
304 parameters
2 restraints
H-atom parameters constrained
Δρ_max_ = 0.19 e Å^−3^
Δρ_min_ = −0.29 e Å^−3^
Absolute structure: Flack (1983 ▶), 1683 Friedel pairs
Flack parameter: 0.42 (11)

### 

Data collection: *SMART* (Bruker, 2001 ▶); cell refinement: *SAINT-Plus* (Bruker, 2001 ▶); data reduction: *SAINT-Plus*; program(s) used to solve structure: *SHELXS97* (Sheldrick, 2008 ▶); program(s) used to refine structure: *SHELXL97* (Sheldrick, 2008 ▶); molecular graphics: *SHELXL97*; software used to prepare material for publication: *SHELXL97*.

## Supplementary Material

Crystal structure: contains datablocks global, I. DOI: 10.1107/S1600536808022253/bh2169sup1.cif

Structure factors: contains datablocks I. DOI: 10.1107/S1600536808022253/bh2169Isup2.hkl

Additional supplementary materials:  crystallographic information; 3D view; checkCIF report

## Figures and Tables

**Fig. 1.**
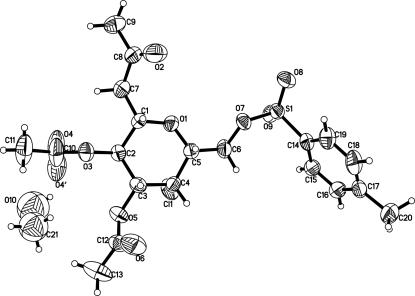
The molecular structure of (I), showing atom displacement ellipsoids drawn at the 50% probability level.

## supplementary crystallographic information

### Comment

Due to their unique chemical and enzymatic hydrolysis stability, C-glycosides are becoming useful building blocks (Postema, 1992) for the total synthesis of various types of natural products such as palytoxin (Lewis *et al.*, 1982), brevetoxin (Nicolaou *et al.*, 1995) and polyether antibiotics (Paterson & Mansuri, 1985), and are used as a model in enzymatic and metabolic studies as well. Despite this attractive applications, structural investigations by using single-crystal X-ray analysis, which provides unambiguous structural data, are rare. Herein we report the design and synthesis of an acylated C-glycosidic analog, which would be of great interest in order to get additional crystallographic information about the substrate.

In the orthorhombic crystals of the title compound, the asymmetric unit contains one molecule and 0.25 methanol solvate. No significant hydrogen bonds exist in the crystal. The value of θ (3.44°) and the magnitude of the torsion angles in the ring (52.9–63.6°) reveal that the ^4^C_1_ pyranose ring presents a slight distortion. The primary hydroxyl group in the title compound is in the gt position [O1—C5—C6—O7 = 74.9 (5)°], which is known to be the favored orientation for pyranose with the *galacto* configuration (Tvaroška *et al.*, 2002).

### Experimental

All reagents were commercially available and of analytical grade. Sulfonylation of 1-(4-chloro-4-deoxy-β-*D*-galactopyranosyl)-propan-2-one with toluene-4-sulfonyl chloride in dry pyridine afforded the 6-toluenesulfonylated intermediate. Further acetylation with acetyl anhydride in pyridine and subsequent purification by chromatography on silica gel furnished the title compound as a white solid. White crystals suitable for X-ray crystallographic analysis were obtained by recrystallization from methanol.

### Refinement

All H atoms bonded to C atoms were positioned geometrically and refined as riding to their parent atoms, with C—H = 0.93–0.98 Å and *U*_iso_(H) = 1.2*U*_eq_(C) or 1.5*U*_eq_(methyl C). For the methanol molecule, the hydroxyl H atom was positioned geometrically and refined as riding with O—H = 0.82Å and *U*_iso_(H) = 1.5*U*_eq_(O). Assuming that starting material is enantiomerically pure, and that anomalous dispersion effects from S and Cl atoms are significant, we suppose that the refined Flack parameter, 0.42 (11) based on 1683 measured Friedel pairs, reflects a partial twinning by merohedry for the sample used for data collection.

### Crystal data

**Table:**

C_20_H_25_ClO_9_S·0.25CH_4_O	*F*_000_ = 1018
*M**_r_* = 484.92	*D*_x_ = 1.347 Mg m^−^^3^
Orthorhombic, *P*2_1_2_1_2_1_	Mo *K*α radiation λ = 0.71073 Å
Hall symbol: P 2ac 2ab	Cell parameters from 399 reflections
*a* = 7.3300 (15) Å	θ = 2–25.1º
*b* = 14.608 (3) Å	µ = 0.29 mm^−^^1^
*c* = 22.329 (5) Å	*T* = 291 (2) K
*V* = 2390.9 (8) Å^3^	Prism, colourless
*Z* = 4	0.20 × 0.17 × 0.16 mm

### Data collection

**Table:**

Bruker SMART APEX CCD area-detector diffractometer	4129 independent reflections
Radiation source: fine-focus sealed tube	3274 reflections with *I* > 2σ(*I*)
Monochromator: graphite	*R*_int_ = 0.052
*T* = 291(2) K	θ_max_ = 25.3º
0.3° wide ω scans	θ_min_ = 1.7º
Absorption correction: multi-scan(SADABS; Bruker, 2001)	*h* = −8→8
*T*_min_ = 0.944, *T*_max_ = 0.954	*k* = −17→17
7242 measured reflections	*l* = −26→26

### Refinement

**Table:**

Refinement on *F*^2^	Hydrogen site location: inferred from neighbouring sites
Least-squares matrix: full	H-atom parameters constrained
*R*[*F*^2^ > 2σ(*F*^2^)] = 0.062	*w* = 1/[σ^2^(*F*_o_^2^) + (0.0873*P*)^2^ + 0.6536*P*] where *P* = (*F*_o_^2^ + 2*F*_c_^2^)/3
*wR*(*F*^2^) = 0.154	(Δ/σ)_max_ < 0.001
*S* = 1.00	Δρ_max_ = 0.19 e Å^−^^3^
4129 reflections	Δρ_min_ = −0.29 e Å^−^^3^
304 parameters	Extinction correction: none
2 restraints	Absolute structure: Flack (1983), 1683 Friedel pairs
Primary atom site location: structure-invariant direct methods	Flack parameter: 0.42 (11)
Secondary atom site location: difference Fourier map	

### Fractional atomic coordinates and isotropic or equivalent isotropic displacement parameters (Å^2^)

**Table:**

	*x*	*y*	*z*	*U*_iso_*/*U*_eq_	Occ. (<1)
S1	0.32977 (15)	0.92085 (8)	0.03975 (5)	0.0411 (3)	
Cl1	0.65974 (18)	1.05079 (11)	0.24709 (5)	0.0662 (4)	
O1	0.7356 (4)	1.1010 (2)	0.11261 (14)	0.0425 (7)	
O2	0.8839 (8)	1.1761 (3)	−0.01365 (18)	0.0918 (16)	
O3	1.1793 (4)	1.1764 (2)	0.16930 (14)	0.0481 (8)	
O4	1.113 (5)	1.3083 (19)	0.2077 (18)	0.054 (11)	0.21 (5)
O4'	1.132 (2)	1.2846 (17)	0.2385 (15)	0.137 (9)	0.79 (5)
O5	1.0691 (4)	1.0491 (2)	0.25895 (13)	0.0512 (8)	
O6	1.2341 (6)	0.9227 (3)	0.2414 (2)	0.0779 (12)	
O7	0.5241 (4)	0.9533 (2)	0.06115 (13)	0.0453 (8)	
O8	0.3338 (5)	0.9324 (2)	−0.02346 (13)	0.0599 (9)	
O9	0.1958 (4)	0.9671 (2)	0.07483 (15)	0.0507 (8)	
O10	1.184 (7)	1.295 (3)	0.3532 (19)	0.205 (15)*	0.25
H10	1.1133	1.2916	0.3250	0.308*	0.25
C1	0.9128 (6)	1.1430 (3)	0.1106 (2)	0.0389 (10)	
H1A	0.9924	1.1071	0.0843	0.047*	
C2	0.9930 (6)	1.1440 (3)	0.17380 (19)	0.0384 (10)	
H2A	0.9211	1.1834	0.2003	0.046*	
C3	1.0018 (6)	1.0463 (3)	0.19808 (18)	0.0400 (10)	
H3A	1.0880	1.0112	0.1736	0.048*	
C4	0.8168 (7)	1.0005 (3)	0.19472 (19)	0.0425 (10)	
H4A	0.8316	0.9356	0.2047	0.051*	
C5	0.7433 (7)	1.0076 (3)	0.1312 (2)	0.0423 (11)	
H5A	0.8257	0.9745	0.1043	0.051*	
C6	0.5551 (7)	0.9680 (4)	0.1250 (2)	0.0528 (13)	
H6A	0.5464	0.9105	0.1466	0.063*	
H6B	0.4649	1.0100	0.1410	0.063*	
C7	0.8895 (7)	1.2381 (3)	0.0847 (2)	0.0525 (12)	
H7A	0.9911	1.2757	0.0979	0.063*	
H7B	0.7790	1.2647	0.1012	0.063*	
C8	0.8787 (7)	1.2427 (4)	0.0170 (2)	0.0544 (13)	
C9	0.8655 (10)	1.3370 (4)	−0.0087 (3)	0.0760 (18)	
H9A	0.8524	1.3331	−0.0514	0.114*	
H9B	0.9742	1.3706	0.0008	0.114*	
H9C	0.7615	1.3677	0.0080	0.114*	
C10	1.2286 (8)	1.2513 (4)	0.1997 (4)	0.0768 (19)	
C11	1.4220 (9)	1.2763 (5)	0.1873 (4)	0.101 (3)	
H11A	1.4464	1.3363	0.2029	0.152*	
H11B	1.4430	1.2759	0.1449	0.152*	
H11C	1.5014	1.2327	0.2063	0.152*	
C12	1.1872 (7)	0.9807 (4)	0.2751 (2)	0.0527 (12)	
C13	1.2456 (10)	0.9929 (6)	0.3384 (3)	0.093 (2)	
H13A	1.3539	0.9577	0.3457	0.139*	
H13B	1.1502	0.9726	0.3647	0.139*	
H13C	1.2705	1.0565	0.3458	0.139*	
C14	0.3198 (6)	0.8038 (3)	0.05653 (18)	0.0417 (10)	
C15	0.2354 (7)	0.7735 (3)	0.1088 (2)	0.0492 (12)	
H15A	0.1912	0.8151	0.1368	0.059*	
C16	0.2191 (8)	0.6808 (3)	0.1182 (2)	0.0557 (13)	
H16A	0.1628	0.6605	0.1531	0.067*	
C17	0.2823 (7)	0.6174 (3)	0.0783 (2)	0.0524 (13)	
C18	0.3687 (7)	0.6498 (4)	0.0270 (3)	0.0593 (14)	
H18A	0.4158	0.6077	−0.0002	0.071*	
C19	0.3868 (7)	0.7414 (4)	0.0151 (2)	0.0547 (13)	
H19A	0.4429	0.7613	−0.0199	0.066*	
C20	0.2580 (10)	0.5161 (4)	0.0872 (3)	0.0769 (18)	
H20A	0.2353	0.5037	0.1288	0.115*	
H20B	0.3666	0.4847	0.0748	0.115*	
H20D	0.1565	0.4952	0.0637	0.115*	
C21	1.292 (6)	1.229 (3)	0.3514 (17)	0.135 (14)*	0.25
H21A	1.2769	1.1924	0.3866	0.202*	0.25
H21B	1.4149	1.2518	0.3496	0.202*	0.25
H21C	1.2672	1.1930	0.3164	0.202*	0.25

### Atomic displacement parameters (Å^2^)

**Table:**

	*U*^11^	*U*^22^	*U*^33^	*U*^12^	*U*^13^	*U*^23^
S1	0.0419 (6)	0.0444 (6)	0.0369 (5)	−0.0053 (5)	−0.0003 (5)	−0.0019 (5)
Cl1	0.0561 (7)	0.1008 (11)	0.0417 (6)	−0.0185 (8)	0.0082 (6)	−0.0121 (6)
O1	0.0387 (16)	0.0413 (18)	0.0476 (18)	−0.0039 (13)	−0.0022 (14)	0.0046 (14)
O2	0.167 (5)	0.053 (2)	0.055 (2)	−0.008 (3)	−0.020 (3)	0.002 (2)
O3	0.0376 (16)	0.0496 (18)	0.0570 (19)	−0.0041 (16)	0.0007 (15)	−0.0052 (15)
O4	0.062 (14)	0.026 (14)	0.07 (2)	−0.006 (9)	−0.013 (12)	−0.011 (12)
O4'	0.086 (6)	0.121 (12)	0.20 (2)	−0.026 (7)	0.017 (10)	−0.107 (14)
O5	0.0522 (18)	0.065 (2)	0.0363 (18)	0.0044 (17)	−0.0092 (14)	−0.0027 (16)
O6	0.078 (3)	0.083 (3)	0.073 (3)	0.026 (2)	−0.012 (2)	−0.003 (2)
O7	0.0475 (17)	0.057 (2)	0.0314 (16)	−0.0150 (16)	0.0000 (13)	−0.0044 (15)
O8	0.069 (2)	0.075 (2)	0.0353 (17)	−0.013 (2)	−0.0064 (16)	0.0054 (15)
O9	0.0485 (18)	0.0431 (18)	0.060 (2)	0.0050 (15)	0.0037 (16)	−0.0022 (15)
C1	0.041 (2)	0.032 (2)	0.044 (3)	−0.0023 (19)	0.0048 (19)	0.005 (2)
C2	0.035 (2)	0.042 (3)	0.039 (2)	0.0004 (19)	0.0023 (18)	−0.002 (2)
C3	0.044 (2)	0.046 (3)	0.030 (2)	0.000 (2)	0.0017 (18)	−0.002 (2)
C4	0.051 (3)	0.037 (2)	0.039 (2)	−0.006 (2)	0.002 (2)	−0.0006 (18)
C5	0.046 (3)	0.043 (3)	0.037 (2)	−0.006 (2)	0.0000 (19)	0.0007 (19)
C6	0.055 (3)	0.061 (3)	0.042 (3)	−0.024 (3)	0.002 (2)	−0.004 (2)
C7	0.058 (3)	0.043 (3)	0.056 (3)	−0.001 (2)	0.000 (2)	−0.002 (2)
C8	0.056 (3)	0.045 (3)	0.062 (3)	−0.008 (2)	−0.009 (2)	0.014 (3)
C9	0.089 (4)	0.054 (3)	0.085 (4)	0.001 (3)	−0.020 (4)	0.027 (3)
C10	0.051 (3)	0.054 (4)	0.125 (6)	−0.007 (3)	−0.004 (4)	−0.033 (4)
C11	0.060 (4)	0.079 (5)	0.166 (8)	−0.026 (3)	−0.006 (4)	−0.015 (5)
C12	0.040 (3)	0.069 (3)	0.049 (3)	0.004 (3)	−0.003 (2)	0.012 (3)
C13	0.089 (5)	0.139 (6)	0.050 (3)	0.041 (5)	−0.013 (3)	0.006 (4)
C14	0.037 (2)	0.048 (2)	0.040 (2)	−0.006 (2)	−0.001 (2)	−0.0071 (19)
C15	0.061 (3)	0.047 (3)	0.040 (3)	0.000 (2)	−0.001 (2)	−0.004 (2)
C16	0.077 (4)	0.044 (3)	0.046 (3)	−0.002 (2)	−0.004 (3)	0.000 (2)
C17	0.056 (3)	0.043 (3)	0.059 (3)	0.001 (2)	−0.021 (3)	−0.006 (2)
C18	0.048 (3)	0.052 (3)	0.077 (4)	0.008 (2)	0.000 (3)	−0.030 (3)
C19	0.051 (3)	0.055 (3)	0.059 (3)	−0.004 (2)	0.010 (2)	−0.019 (3)
C20	0.090 (4)	0.041 (3)	0.099 (5)	0.003 (3)	−0.032 (4)	−0.003 (3)

### Geometric parameters (Å, °)

**Table:**

S1—O8	1.422 (3)	C7—H7A	0.9700
S1—O9	1.427 (3)	C7—H7B	0.9700
S1—O7	1.576 (3)	C8—C9	1.496 (7)
S1—C14	1.753 (5)	C9—H9A	0.9600
Cl1—C4	1.798 (5)	C9—H9B	0.9600
O1—C5	1.426 (5)	C9—H9C	0.9600
O1—C1	1.437 (5)	C10—C11	1.490 (8)
O2—C8	1.189 (7)	C11—H11A	0.9600
O3—C10	1.337 (6)	C11—H11B	0.9600
O3—C2	1.449 (5)	C11—H11C	0.9600
O4—C10	1.20 (4)	C12—C13	1.488 (8)
O4'—C10	1.222 (16)	C13—H13A	0.9600
O5—C12	1.370 (6)	C13—H13B	0.9600
O5—C3	1.447 (5)	C13—H13C	0.9600
O6—C12	1.184 (6)	C14—C19	1.388 (6)
O7—C6	1.459 (6)	C14—C15	1.393 (6)
O10—C21	1.24 (5)	C15—C16	1.375 (7)
O10—H10	0.8200	C15—H15A	0.9300
C1—C7	1.515 (6)	C16—C17	1.367 (7)
C1—C2	1.530 (6)	C16—H16A	0.9300
C1—H1A	0.9800	C17—C18	1.392 (7)
C2—C3	1.527 (6)	C17—C20	1.503 (8)
C2—H2A	0.9800	C18—C19	1.371 (8)
C3—C4	1.514 (7)	C18—H18A	0.9300
C3—H3A	0.9800	C19—H19A	0.9300
C4—C5	1.521 (6)	C20—H20A	0.9600
C4—H4A	0.9800	C20—H20B	0.9600
C5—C6	1.503 (6)	C20—H20D	0.9600
C5—H5A	0.9800	C21—H21A	0.9600
C6—H6A	0.9700	C21—H21B	0.9600
C6—H6B	0.9700	C21—H21C	0.9600
C7—C8	1.516 (7)		
			
O8—S1—O9	120.2 (2)	C8—C9—H9B	109.5
O8—S1—O7	104.3 (2)	H9A—C9—H9B	109.5
O9—S1—O7	108.28 (18)	C8—C9—H9C	109.5
O8—S1—C14	109.2 (2)	H9A—C9—H9C	109.5
O9—S1—C14	108.4 (2)	H9B—C9—H9C	109.5
O7—S1—C14	105.4 (2)	O4—C10—O4'	37.7 (13)
C5—O1—C1	112.4 (3)	O4—C10—O3	116.9 (17)
C10—O3—C2	119.2 (4)	O4'—C10—O3	122.0 (8)
C12—O5—C3	116.3 (4)	O4—C10—C11	121.9 (16)
C6—O7—S1	118.8 (3)	O4'—C10—C11	125.9 (8)
C21—O10—H10	109.5	O3—C10—C11	111.3 (6)
O1—C1—C7	107.6 (4)	C10—C11—H11A	109.5
O1—C1—C2	108.8 (3)	C10—C11—H11B	109.5
C7—C1—C2	112.7 (4)	H11A—C11—H11B	109.5
O1—C1—H1A	109.2	C10—C11—H11C	109.5
C7—C1—H1A	109.2	H11A—C11—H11C	109.5
C2—C1—H1A	109.2	H11B—C11—H11C	109.5
O3—C2—C3	106.9 (3)	O6—C12—O5	122.5 (5)
O3—C2—C1	107.6 (3)	O6—C12—C13	127.3 (5)
C3—C2—C1	109.5 (3)	O5—C12—C13	110.1 (5)
O3—C2—H2A	110.9	C12—C13—H13A	109.5
C3—C2—H2A	110.9	C12—C13—H13B	109.5
C1—C2—H2A	110.9	H13A—C13—H13B	109.5
O5—C3—C4	111.4 (3)	C12—C13—H13C	109.5
O5—C3—C2	108.8 (3)	H13A—C13—H13C	109.5
C4—C3—C2	111.0 (4)	H13B—C13—H13C	109.5
O5—C3—H3A	108.6	C19—C14—C15	120.5 (4)
C4—C3—H3A	108.6	C19—C14—S1	118.9 (4)
C2—C3—H3A	108.6	C15—C14—S1	120.5 (3)
C3—C4—C5	109.5 (4)	C16—C15—C14	118.6 (5)
C3—C4—Cl1	111.1 (3)	C16—C15—H15A	120.7
C5—C4—Cl1	110.6 (3)	C14—C15—H15A	120.7
C3—C4—H4A	108.5	C17—C16—C15	122.6 (5)
C5—C4—H4A	108.5	C17—C16—H16A	118.7
Cl1—C4—H4A	108.5	C15—C16—H16A	118.7
O1—C5—C6	107.8 (4)	C16—C17—C18	117.5 (5)
O1—C5—C4	110.5 (4)	C16—C17—C20	122.7 (6)
C6—C5—C4	112.7 (4)	C18—C17—C20	119.8 (5)
O1—C5—H5A	108.6	C19—C18—C17	122.3 (4)
C6—C5—H5A	108.6	C19—C18—H18A	118.8
C4—C5—H5A	108.6	C17—C18—H18A	118.8
O7—C6—C5	106.9 (4)	C18—C19—C14	118.6 (5)
O7—C6—H6A	110.4	C18—C19—H19A	120.7
C5—C6—H6A	110.4	C14—C19—H19A	120.7
O7—C6—H6B	110.4	C17—C20—H20A	109.5
C5—C6—H6B	110.4	C17—C20—H20B	109.5
H6A—C6—H6B	108.6	H20A—C20—H20B	109.5
C1—C7—C8	115.2 (4)	C17—C20—H20D	109.5
C1—C7—H7A	108.5	H20A—C20—H20D	109.5
C8—C7—H7A	108.5	H20B—C20—H20D	109.5
C1—C7—H7B	108.5	O10—C21—H21A	109.5
C8—C7—H7B	108.5	O10—C21—H21B	109.5
H7A—C7—H7B	107.5	H21A—C21—H21B	109.5
O2—C8—C9	122.3 (5)	O10—C21—H21C	109.5
O2—C8—C7	122.5 (5)	H21A—C21—H21C	109.5
C9—C8—C7	115.2 (5)	H21B—C21—H21C	109.5
C8—C9—H9A	109.5		
			
O8—S1—O7—C6	167.7 (4)	O1—C5—C6—O7	74.9 (5)
O9—S1—O7—C6	38.6 (4)	C4—C5—C6—O7	−162.9 (4)
C14—S1—O7—C6	−77.2 (4)	O1—C1—C7—C8	−81.7 (5)
C5—O1—C1—C7	174.6 (4)	C2—C1—C7—C8	158.4 (4)
C5—O1—C1—C2	−63.0 (5)	C1—C7—C8—O2	1.7 (8)
C10—O3—C2—C3	−121.1 (5)	C1—C7—C8—C9	−177.0 (5)
C10—O3—C2—C1	121.3 (5)	C2—O3—C10—O4	−31 (2)
O1—C1—C2—O3	173.3 (3)	C2—O3—C10—O4'	12 (2)
C7—C1—C2—O3	−67.5 (5)	C2—O3—C10—C11	−177.5 (5)
O1—C1—C2—C3	57.5 (4)	C3—O5—C12—O6	0.8 (7)
C7—C1—C2—C3	176.7 (4)	C3—O5—C12—C13	179.5 (5)
C12—O5—C3—C4	96.5 (4)	O8—S1—C14—C19	25.0 (5)
C12—O5—C3—C2	−140.9 (4)	O9—S1—C14—C19	157.6 (4)
O3—C2—C3—O5	66.7 (4)	O7—S1—C14—C19	−86.6 (4)
C1—C2—C3—O5	−177.1 (3)	O8—S1—C14—C15	−151.0 (4)
O3—C2—C3—C4	−170.5 (3)	O9—S1—C14—C15	−18.4 (4)
C1—C2—C3—C4	−54.3 (5)	O7—S1—C14—C15	97.4 (4)
O5—C3—C4—C5	174.4 (4)	C19—C14—C15—C16	−0.5 (7)
C2—C3—C4—C5	53.1 (5)	S1—C14—C15—C16	175.4 (4)
O5—C3—C4—Cl1	52.0 (5)	C14—C15—C16—C17	0.0 (8)
C2—C3—C4—Cl1	−69.4 (4)	C15—C16—C17—C18	1.1 (8)
C1—O1—C5—C6	−173.7 (4)	C15—C16—C17—C20	−177.5 (5)
C1—O1—C5—C4	62.8 (5)	C16—C17—C18—C19	−1.8 (8)
C3—C4—C5—O1	−56.3 (5)	C20—C17—C18—C19	176.8 (5)
Cl1—C4—C5—O1	66.5 (4)	C17—C18—C19—C14	1.3 (8)
C3—C4—C5—C6	−176.9 (4)	C15—C14—C19—C18	−0.2 (7)
Cl1—C4—C5—C6	−54.2 (5)	S1—C14—C19—C18	−176.2 (4)
S1—O7—C6—C5	−174.1 (3)		
